# Multi-Level Cross-Modal Interactive-Network-Based Semi-Supervised Multi-Modal Ship Classification

**DOI:** 10.3390/s24227298

**Published:** 2024-11-15

**Authors:** Xin Song, Zhikui Chen, Fangming Zhong, Jing Gao, Jianning Zhang, Peng Li

**Affiliations:** The School of Software Technology, Dalian University of Technology, Dalian 116621, China; songxin72@mail.dlut.edu.cn (X.S.);

**Keywords:** ship classification, deep multi-modal learning, semi-supervised learning

## Abstract

Ship image classification identifies the type of ships in an input image, which plays a significant role in the marine field. To enhance the ship classification performance, various research focuses on studying multi-modal ship classification, which aims at combining the advantages of visible images and infrared images to capture complementary information. However, the current methods simply concatenate features of different modalities to learn complementary information, which neglects the multi-level correlation between different modalities. Moreover, the existing methods require a large amount of labeled ship images to train the model. How to capture the multi-level cross-modal correlation between unlabeled and labeled data is still a challenge. In this paper, a novel semi-supervised multi-modal ship classification approach is proposed to solve these issues, which consists of two components, i.e., multi-level cross-modal interactive network and semi-supervised contrastive learning strategy. To learn comprehensive complementary information for classification, the multi-level cross-modal interactive network is designed to build local-level and global-level cross-modal feature correlation. Then, the semi-supervised contrastive learning strategy is employed to drive the optimization of the network with the intra-class consistency constraint based on supervision signals of unlabeled samples and prior label information. Extensive experiments on the public datasets demonstrate that our approach achieves state-of-the-art semi-supervised classification effectiveness.

## 1. Introduction

Ship classification has aroused increasing attention and played a significant role in the marine field, such as combating illegal fishing and managing maritime traffic. With the development of Earth observation technology, ship images collected by the maritime surveillance system have surged, which makes ship classification more attractive for relevant applications. At present, a great deal of research focuses on ship image classification, which utilizes the characteristics extracted from the input image to recognize the ship category [1,2,3].

Mainstream ship images for classification contain synthetic aperture radar (SAR) images, visible images, and infrared images [4,5,6]. SAR images are obtained from radars and are vulnerable to other electromagnetic interference, which can only distinguish large vessels from the background [7,8,9]. Compared with SAR images, visible images have higher resolution and provide more texture details for classifying fine-grained ship targets and have attracted great attention from researchers [10]. Visible images are generated by reflected light and can capture abundant visual details, while they cannot capture explicit ship target information under poor lighting conditions. Infrared images can ameliorate the problem by providing complementary ship target information for visible images of the same scene. The multi-modal ship image (visible and infrared image) classification combines the advantages of visible images and infrared images to mine the complementary information for classification, which has demonstrated significant advantages over single-modal images.

Traditional multi-modal methods manually incorporate hand-crafted features of different modalities into common representations to learn complementary information and then feed common representations to a classifier like SVM for classification [11]. It is noted that these methods depend heavily on image quality and show poor classification performance in complex imaging conditions, where illumination or viewing angles change significantly. To solve this problem, deep-learning methods are proposed to automatically extract deep features of different modalities through convolutional neural networks and then concatenate these features to capture complementary information. Then, like traditional methods, deep-learning methods feed concatenated deep common representations into a classifier. For example, Ren et al. [10] concatenates high-level features extracted by CNN and spatial location features extracted by Hu invariant moments [12] as common representations for ship image classification.

Despite the success of multi-modal ship classification, the current network-based methods usually simply concatenate features of different modalities, which ignores the correlation between different modalities. However, capturing the correlation between different modalities from texture and semantic perspectives is critical for learning comprehensive complementary information about ship images. Furthermore, all the above methods have the common drawback that the training progress requires a large amount of labeled ship images [13,14,15]. The existing methods neglect that the number of labeled ship images is usually limited and cannot utilize unlabeled data effectively, which can pose a great difficulty for model training. For the current semi-supervised multi-modal ship classification methods, how to make full use of the cross-modal correlation implied in unlabeled and labeled data has not been well studied.

**Figure 1:**
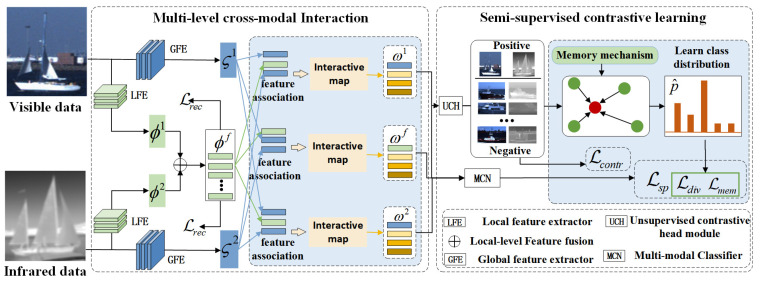
Framework of the proposed approach. Given a batch of multi-modal images, the approach utilizes the multi-level cross-modal interactive network to encode deep correlated features $\omega^{i}$ of multi-modal data through mining the correlation among global-level features $\varsigma^{i}$ for each modal data and local-level fused features $\phi^{f}$. $\phi^{f}$ are based on the fusion between texture features $\phi^{i}$ of each modal datum through the spatial and channel attention mechanism. For the optimization of the network, the self-supervised contrastive learning strategy achieves the intra-class consistency constraint based on class distribution $\varpi^{f}$ of unlabeled data generated through the memory mechanism and prior label information, which contains unsupervised contrastive loss $L_{contr}$ and semi-supervised divergence loss $\left(L_{div},L_{mem}\right)$. In addition, A reconstructive loss $L_{rec}$ is used to guide the model pre-training. A supervised loss $L_{sp}$ is used to learn label information. Contrastive representations $\varpi^{i}$ are outputs of the unsupervised contrastive head module, which are the input of the memory mechanism.

In this paper, a novel semi-supervised multi-modal ship classification approach (Semi-MMSC) is proposed (see Figure 1) that utilizes the multi-level cross-modal interactive network to learn comprehensive complementary information and introduces a novel semi-supervised contrastive learning strategy to guide the optimization of the network. The multi-level cross-modal interactive network mines the correlation of different modalities in both local-level and global-level branches. In the local-level branch, to model the relationship between different modalities from the texture perspective, Semi-MMSC first utilizes local feature extractors to obtain texture features of different modalities. Then, the interactive network employs the local-level fusion module to fuse these texture features with the spatial and channel attention mechanism. In the global-level branch, Semi-MMSC extracts global features of different modalities first. After that, to capture the relationship between different modalities from the semantic perspective, the network builds the feature interactive map among global-level features with local-level fused features as a medium via the global-level interactive module.

To alleviate the influence of label scarcity on model training, the semi-supervised contrastive learning strategy incorporates contrastive loss into the semi-supervised learning and optimizes the network with these two loss functions. Specifically, with the unsupervised contrastive loss, the contrastive representations are encoded by maximizing the similarity between correlated features of the same sample, which can learn similarity distribution from data and be used as supervised signals of unlabeled data. To train the network with obtained supervision signals of unlabeled data, the memory mechanism is designed to generate class prototypes based on contrastive representations. Then, the self-supervised loss function is designed to constrain the intra-class consistency between unlabeled and labeled multi-modal data based on obtained supervision signals and prior label information. The experimental results on the public datasets confirm that the proposed approach outperforms the state-of-the-art semi-supervised models in terms of classification performance.

The main contributions of this paper can be summarized as follows:

1. The multi-level cross-modal interactive network is proposed to implement local-level and global-level cross-modal feature interaction, which encodes deep correlated features containing more comprehensive complementary information for classification.
2. The semi-supervised contrastive learning strategy is designed to derive supervised signals from unlabeled samples with contrastive learning and learn the intra-class consistency constraint between unlabeled and labeled data to optimize the model.
3. The performance comparison shows the enhanced classification performance of the proposed approach over the current state-of-the-art on the public datasets.

## 2. Related Works

### 2.1. Ship Classification

Ship classification methods extract visual characteristics from input sensors and then utilize these characteristics to recognize which categories the input belongs to, which play a crucial role in many marine tasks, such as vessel management and navigation safety protection.

**Single-modal Ship Classification:** The traditional methods attempt to learn visual characteristics only based on the single image source, including SAR data, infrared data, and visible data. SAR is one kind of active microwave imaging sensor that can collect all-day and all-weather data [16,17]. Wang et al. [18] designed a hierarchical ship classifier for COSMO-SkyMed SAR images where the geometric and backscattering characteristics of various ship types were analyzed and used. Wu et al. [19] proposed a ship classification method based on high-resolution SAR images to distinguish bulk carriers, container ships, and oil tankers. The method first designed one three-element feature vector that was composed of kernel density mean features, three structural features, and a backscattering coefficient estimation mean feature, and then, this feature vector was input into one SVM classifier for the final ship classification. Li et al. [2] proposed a clustering-based size-adaptive safer oversampling technique for SAR ship classification that selects minority samples adaptively and then generates new instances with the safe metric to alleviate the imbalanced dataset problem. Generally, SAR images are used for distinguishing differences between large-size ships due to the limitation of image resolution.

On the contrary, visible images and infrared images can provide richer visual details and explicit texture information for classification that attract a large amount of attention. Visible images are generated by the visible sensor capturing reflected light and contain rich texture information while being sensitive to illumination changes [20,21,22]. Infrared images are generated by the infrared sensor capturing thermal radiations and contain rich target information by presenting the different distributions of temperature in the scene [23,24,25]. However, infrared images contain little texture information due to the problem of low resolution and blurred edges. To complement the deficiency of each modal datum, various researchers have attempted to study multi-modal ship classification, which captures complementary information hidden in multi-modal data for ship classification.

**Multi-modal Ship Classification:** Recently, multi-modal ship classification methods have been proposed that extract generic features from different modalities and combine these features to learn cross-modal complementary information hidden in the data. Traditional multi-modal methods utilize hand-crafted feature extractions to extract features from the data. The common feature extractions include the histogram of oriented gradients (HOG) [26], scale-invariant feature transform (SIFT) [27], local binary patterns (LBP) [28], etc. Then, a classification model like support vector machine (SVM) is used to classify images based on the obtained hand-crafted features. For example, because HOG features have better representation ability than other hand-crafted features, Lin et al. [28] proposed to utilize the manifold learning SAR-HOG features to classify ship objects. However, these hand-crafted features can only learn low-level visual characteristics, and the extraction process consumes a large amount of human effort. More importantly, traditional multi-modal methods have high requirements for image quality, which are impractical for ship classification since the actual data collection environment is complex.

To solve the above problems, many deep-learning methods are proposed to adopt deep convolutional networks as the base architecture to extract deep features automatically, which can learn high-level features from images. These high-level features are more invariant than low-level visual features, which are important for distinguishing the differences in different classes. For example, Zhang et al. [1] proposed to combine HOG features and deep CNN features with the hog feature fusion mechanism to improve the classification performance. To both preserve the internal structure of the category information and select the minimal dimension features, the feature fusion architecture based on spectral regression discriminant analysis is built to combine features from infrared and visible images [29]. Ren et al. [10] proposed a hybrid method that fuses high-level features extracted by CNN and spatial location features extracted by Hu invariant moments for ship image classification. Although the current multi-modal ship classification methods have achieved significant success, the application of deep learning methods is still limited due to the expensive cost of massive labeled data collection. To solve this problem, some works attempt to introduce semi-supervised learning into the maritime field [30,31,32].

### 2.2. Semi-Supervised Learning

Semi-supervised learning focuses on utilizing a few labeled data and a large amount of unlabeled data to implement the classification task, which has been a long-standing problem in computer vision. The recent self-supervised methods consist of two branches: pseudo-label-based methods and consistency-regularization-based methods. The former focuses on producing pseudo labels based on a pre-trained model for unlabeled samples and then training the model with these pseudo labels [33,34]. The whole training process is viewed as a self-training process, which suffers the overfitting problem caused by incorrect pseudo labels. lscen et al. [34] made a detour by the label propagation to spread labeled data distribution on unlabeled data. Consistency-regularization-based methods aim to produce consistent predictions on different image views of the same image and then adopt the pseudo-label-based consistency learning strategy to train the model [35,36,37]. The previous methods created different views of an image by strong augmentations to simulate image perturbations of an object. At present, pseudo-label-based consistency training, which uses a weak augmentation for creating pseudo labels and a strong augmentation for consistency training, has dominated and achieved excellent performance gain.

In the ship classification task, some methods try to transfer learned prior class knowledge into the training of unlabeled ship images to achieve better classification results. For example, Yang et al. [30] transfers automatic identification system (AIS) knowledge to train the network on the image domain, which extracts static ship information from AIS data to assist in learning ship category information. However, there is no literature for semi-supervised multi-modal ship classification, which aims at dealing with the classification problem of unlabeled multi-modal ship data. Hence, we propose a novel approach, which can make use of cross-modal complementary information from unlabeled data and limited labeled data for multi-modal ship classification.

## 3. The Proposed Approach

In this section, details of the proposed approach are introduced, including the preliminaries, the network architecture, and loss functions.

### 3.1. Preliminaries

The multi-modal ship dataset is defined as $D=\{X^{1},\ldots ,X^{M}\},M\;=2$, where *M* denotes the number of the modalities. $X^{m}={\left\{x_{i}^{m}\in R^{C\times H\times W}\right\}}_{i=1}^{N}$ denotes *m*-th modal data. *N* denotes the number of datasets, *C* denotes the image channel, *H* and *W*, respectively, denote the image height and width. Two ResNets are adopted as the global feature extractor to extract deep global-level features $\varsigma^{m}\in R^{N_{g}}$ of different modalities, $\varsigma^{m}=F^{m}\left(X^{m};W^{m}\right),m\in 1,\ldots ,M$, where $F^{m}$ represents the *m*-th convolutional network and $W^{m}$ denotes the corresponding network weights, respectively. $N_{g}$ denotes the global-level feature dimension.

### 3.2. Multi-Level Cross-Modal Interactive Network

Concatenating deep global-level features of these two modalities linearly cannot learn comprehensive complementary information hidden in unlabeled data. To alleviate this problem, the multi-level cross-modal interactive network is proposed to mine the feature correlation of different modalities in the local-level and global-level branches.

#### 3.2.1. Local-Level Fusion Module

In the local branch, to build the correlation of texture features, the network first employs the local feature extractor to extract texture features from images of different modal data and then combines these features with the spatial and channel attention mechanism. The local-level fusion module consists of two stages, extraction and fusion. To learn the texture information hidden in data with the local-level fusion module, the reconstruct loss is designed to train the module, which is demonstrated in the following sections.

**Extraction:** For the input image of each model, the local-level fusion module adopts the local feature extractor to extract texture features of *i*-th modal data ${\phi_{l}}^{i}\in R^{C_{l}\times H_{l}\times W_{l}}$. $C_{l}$ denotes the number of texture feature channels at the *l*-th layer. $H_{l}$ and $W_{l}$ denote the number of the texture feature height and width at the *l*-th layer. The local feature extractor consists of four convolutional blocks. Each convolutional block contains max-pool and stride convolution. These operations are used as downsample operations to maintain texture information in the feature extraction process. The whole network architecture is shown in Table 1.

**Fusion:** To obtain local-level fused features based on texture features of different modalities, the local-level fusion module performs the spatial and channel attention mechanism on texture features to generate the spatial and channel attention maps, respectively, and then fuses two attention maps. Specifically, the spatial attention mechanism calculates the spatial attention map by the weighted sum of the features of different modes along the spatial dimension ${\phi_{l}}^{p}\in R^{C_{l}\times H_{l}\times W_{l}}$

(1) $$\begin{array}{c} {\phi_{l}}^{p}=\sum_{j=1}^{M}\frac{{∥{\phi_{l}}^{j}\left(a,b\right)∥}_{1}}{{\sum_{i=1}^{M}∥{\phi_{l}}^{i}\left(a,b\right)∥}_{1}}\times {\phi_{l}}^{j}\left(a,b\right) \end{array}$$

where ${∥\cdot ∥}_{1}$ denotes $l_{1}$-norm, *M* denotes the number of the modalities. ${\phi_{l}}^{i}\left(a,b\right)$ indicates features of the *i*-th modal at the pixel position (a,b). Similarly, the local-level fusion module utilizes the channel attention mechanism to calculate the channel attention map by the weighted sum of features of different modalities along the channel dimension ${\phi_{l}}^{c}\in R^{C_{l}\times H_{l}\times W_{l}}$

(2) $$\begin{array}{c} {\phi_{l}}^{c}=\sum_{j=1}^{M}\frac{P({\phi_{l}}^{j}\left(c\right))}{\sum_{i=1}^{M}P({\phi_{l}}^{i}\left(c\right))}\times {\phi_{l}}^{j}\left(c\right) \end{array}$$

where *P* denotes the global pooling operator, ${\phi_{l}}^{i}\left(c\right)$ indicates features of the *i*-th modal at the channel *c*. Local-level fused features are obtained by combining the spatial attention map and the channel attention map ${\phi_{l}}^{f}=\left({\phi_{l}}^{p}+{\phi_{l}}^{c}\right)\times \frac{1}{2}$.

#### 3.2.2. Global-Level Interactive Module

**Figure 2:**
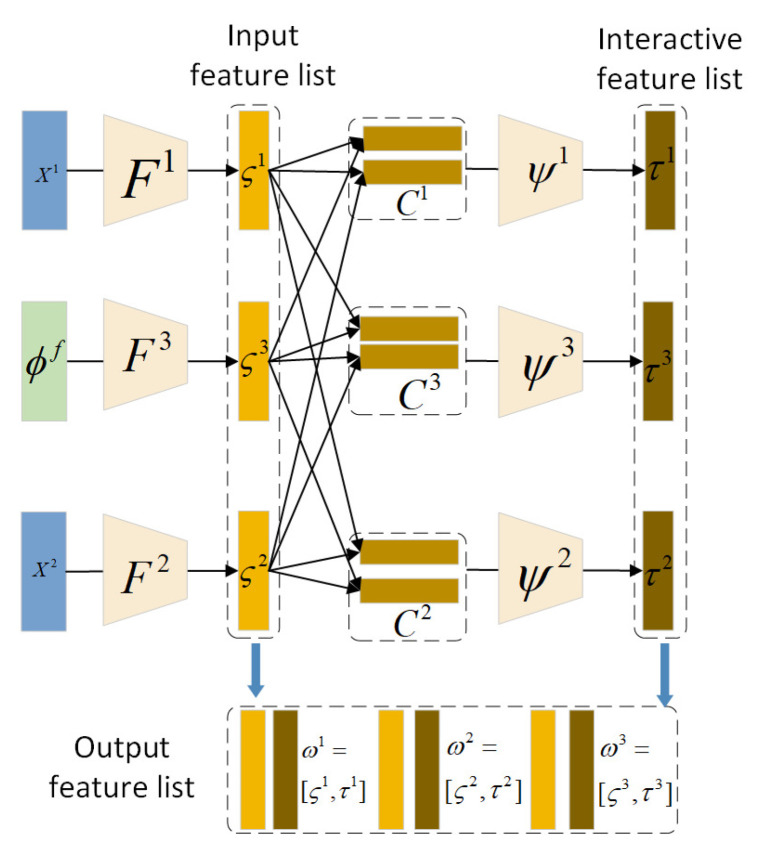
Framework of the proposed global-level interactive module.

As shown in Figure 2, in the global-level branch, the global-level interactive module aims at building the feature interactive map between global-level features of visible and infrared data with local-level fused features as a medium, which encodes correlated features that contain deep complementary information. First, we define the input feature list of the global-level interactive module as

(3) $$\begin{array}{c} \begin{array}{c} InputFeatureList=[\varsigma^{1},\ldots ,\varsigma^{M},\varsigma^{M+1}],\\ where\;\varsigma^{M+1}=F^{m+1}\left({\phi_{1}}^{f};W^{M+1}\right)\in R^{N_{g}} \end{array} \end{array}$$

Although there are four layers of local-level fused features obtained by the local-level fusion module, this paper only adopts features of the first layer as the input feature because features ${\phi_{1}}^{f}$ can contain more comprehensive texture information than other layers. For each pair of features, the pairwise interactive matrix is first computed by performing matrix multiplication on two feature vectors

(4) $$\begin{array}{c} \gamma \left(i,\bar{i}\right)=\varsigma^{i}\varsigma^{{\bar{i}}^{T}},\;where\;i\in \left[1,M+1\right] \end{array}$$

where $\bar{i}=\left\{1,\ldots ,M+1\right\}\setminus i$. Then, the feature interactive map of the *i*-th input with respect to other feature vectors is defined as follows:

(5) $$\begin{array}{c} C^{i}=\sum_{\left(i,\bar{i}\right)\in S^{i}}\gamma (i,\bar{i}) \end{array}$$

where $C^{i}$ denotes the vectorization of the matrix, and $S^{i}={\left\{\left(\varsigma^{i},\varsigma^{\bar{i}}\right)\right\}}_{\bar{i}=\left\{1,\ldots ,M+1\right\}\setminus i}$ denotes the sets of feature pairs. The feature interactive map builds the correlations among global-level features of different modalities and local-level fused features, and models the inter-modal relationship from the semantic perspective.

The above feature interactive map needs to calculate the feature vectors of all pairs, which may cause feature redundancy. The global-level interactive module further reduces the feature dimension through a multi-layer fully connected network $\psi^{i}$

(6) $$\begin{array}{c} \tau^{i}=\psi^{i}\left(C^{i};W^{\psi }\right) \end{array}$$

where $W^{\psi }\in R^{N_{g}\times N_{I}}$ denotes the network weights. Then, the deep correlated features are generated by combining interactive features and input features of the global-level interactive module $\omega^{i}=\left[\varsigma^{i},\tau^{i}\right]\in R^{N_{g}+N_{I}}$.

### 3.3. Semi-Supervised Contrastive Learning Strategy

After obtaining deep correlated features of the unlabeled data that can provide rich complementary information, the approach needs to further alleviate the influence of label scarcity. Toward this goal, the semi-supervised contrastive correlation learning strategy is proposed, which comprises two major components: unsupervised contrastive loss and class-wise memory loss.

#### 3.3.1. Unsupervised Contrastive Loss

Contrastive learning utilizes different correlated features from the same image as positive samples and other samples from the training batch as negative samples, which enforces the similarity of correlated features between different modalities and produces contrastive representations to learn complementary information from unlabeled data. Specifically, for correlated features of the *i*-th sample belonging to two modalities $\left({\omega_{i}}^{1},{\omega_{i}}^{2},{\omega_{i}}^{3}\right)$, the contrastive head module first multiplies the correlated features by element to produce contrastive representations, which learn consensus information of the correlated features between different modalities. The contrastive representations of a sample contain three groups, ${\varpi_{i}}^{1}=∥{\omega_{i}}^{2}∥∥{\omega_{i}}^{3}∥,{\varpi_{i}}^{2}=∥{\omega_{i}}^{1}∥∥{\omega_{i}}^{3}∥,{\varpi_{i}}^{3}=∥{\omega_{i}}^{1}∥∥{\omega_{i}}^{2}∥$. Then, the contrastive head module measures the similarity of positive samples, $sim\left({\varpi_{i}}^{1}\right)=\frac{{\left({\omega_{i}}^{2}\right)}^{T}{\omega_{i}}^{3}}{{\varpi_{i}}^{1}},sim\left({\varpi_{i}}^{2}\right)=\frac{{\left({\omega_{i}}^{1}\right)}^{T}{\omega_{i}}^{3}}{{\varpi_{i}}^{2}},sim\left({\varpi_{i}}^{3}\right)=\frac{{\left({\omega_{i}}^{1}\right)}^{T}{\omega_{i}}^{3}}{{\varpi_{i}}^{3}}$.

To encourage contrastive representations can learn model consensus information from correlated features, the corresponding unsupervised contrastive loss for all positive pairs of the *i*-th sample is formulated as

(7) $$\begin{array}{c} Contr_{i}=-\frac{1}{M+1}\sum_{m=1}^{M+1}\log\frac{\exp(sim\left({\varpi_{i}}^{m}\right))/\nu }{\sum_{k=1}^{N}(exp\left(sim\_neg\left({\varpi_{i}}^{m}\right)\right))/\nu } \end{array}$$

where ν denotes the temperature parameter. The negative samples are denoted as $sim\_neg\left({\varpi_{i}}^{1}\right)=\frac{{\left({\omega_{i}}^{2}\right)}^{T}{\omega_{k}}^{3}}{∥{\omega_{i}}^{2}∥∥{\omega_{k}}^{3}∥},sim\_neg\left({\varpi_{i}}^{2}\right)=\frac{{\left({\omega_{i}}^{1}\right)}^{T}{\omega_{k}}^{3}}{∥{\omega_{i}}^{1}∥∥{\omega_{k}}^{3}∥},sim\_neg\left({\varpi_{i}}^{3}\right)=\frac{{\left({\omega_{i}}^{1}\right)}^{T}{\omega_{k}}^{2}}{∥{\omega_{i}}^{1}∥∥{\omega_{k}}^{2}∥}$. By minimizing $L_{contr}=\sum_{i=1}^{N}Contr_{i}$, correlated features of the same sample become more similar, and correlated features of the different samples become more dissimilar. In consequence, contrastive representations of similar samples have large values, and contrastive representations of dissimilar samples have small values.

For reducing computational complexity, this paper only adopts $\varpi^{f}={\varpi_{i}}^{2}$ instead of all pairs of contrastive representations to generate class prototypes of the *i*-th sample. Specifically, the memory mechanism takes the key-value structure as the backbone, which learns class-wise prototypical features from contrastive representations of the whole dataset and predicts class probability for unlabeled data. Key embedding stores the prototypical feature of each class and the value embedding records the probabilistic prediction of the same class.

First, the memory mechanism utilizes contrastive representations of labeled data to update the key and value embedding.

(8) $$\begin{array}{c} \begin{array}{c} k_{j}=k_{j}-\eta \frac{\sum_{i=1}^{N_{j}}\left(k_{j}-\varpi_{i}^{f}\right)}{1+N_{j}},\\ v_{j}=\frac{v_{j}-\eta \Delta_{j}}{{\sum_{i=1}^{K}v_{j,i}-\eta \Delta }_{j,i}},where\;\Delta_{j}=\frac{\sum_{i=1}^{N_{j}}v_{j}-p_{i}}{1+N_{j}} \end{array} \end{array}$$

where η denotes the learning rate, *K* denotes the number of classes, and $N_{j}$ denotes the number of samples from the *j*-th class (j∈{1,…,K}). $p_{i}$ indicates the network prediction of the sample *i*. Along with the training progress, the key and value embeddings become more reliable to reflect the class distribution. At the beginning, the key and value are initialized to 0 and $\frac{1}{K}$, respectively.

With these key and value embeddings, class-wise prototypical features of the labeled data are preserved and the class-wise memory prediction of a sample $x_{i}$ is calculated ${\hat{p}}_{i}=\sum_{i=1}^{K}w\left(k_{i},v_{i}|I\right)\varpi_{i}^{f}$, where $w(k_{i},v_{i}|x_{i})$ denotes the class probability. Specifically, for labeled samples, the class probability is based on the label of the sample. For unlabeled samples, the class probability is based on the pairwise similarity between the contrastive representations $\varpi^{f}$ and the key embedding $k_{i}$

(9) $$\begin{array}{c} w\left(k_{i},v_{i}|x_{i}\right)=\frac{e^{-dist(\varpi^{f},k_{i})}}{\sum_{j=1}^{K}e^{-dist(\varpi^{f},k_{j})}} \end{array}$$

#### 3.3.2. Class-Wise Memory Loss

Based on class-wise memory predictions of the unsupervised data, the semi-supervised loss function is formulated for the optimization of training, which ensures the class-wise consistency between unlabeled and labeled data. The loss function contains two parts, the class divergence loss and the memory entropy loss. The class divergence loss encourages the consistency of class information between the memory module and the network while the memory entropy loss is to encourage the memory module to learn discriminative class information.

The class divergence loss aims to expand class information hidden in the memory module into the training of the unlabeled data.

(10) $$\begin{array}{c} L_{div}=\sum_{j=1}^{K}p\left(j\right)log\frac{p(j)}{\hat{p}\left(j\right)} \end{array}$$

The class divergence loss penalizes the discrepancy between two distributions, p(j) and $\hat{p}\left(j\right)$. When $L_{div}\to 0$, it indicates the network predictions based on correlated features match well with memory predictions.

To penalize inconsistent predictions, the memory entropy loss is designed, which is motivated by the entropy minimization principle,

(11) $$\begin{array}{c} L_{mem}=-\sum_{j=1}^{K}\hat{p}\left(j\right)\log\hat{p}\left(j\right) \end{array}$$

The memory entropy loss reflects the overall memory inference uncertainty and penalizes uncertain memory predictions. A high value of the loss indicates that the memory cannot easily distinguish classes of ship images, which results from assigning inconsistent probabilistic predictions to correlated features within the same class.

### 3.4. Model Training

In this subsection, the whole progress of the network training is introduced. Before training the whole network, the local-level fusion module needs to be pre-trained, which enforces that the texture information hidden in the data can be captured by local-level fused features. Specially, the pre-trained reconstruct loss is defined as follows:

(12) $$\begin{array}{c} L_{rec}=L_{pix}+L_{str} \end{array}$$

where $L_{pix}$ and $L_{str}$ indicate the pixel loss and structure loss, respectively. The pixel loss calculates the distance between input images and the reconstructed images $X^{rec}$, which makes sure the reconstructed images are more similar to the input images

(13) $$\begin{array}{c} L_{pix}=\frac{1}{2}\times \left({∥X^{rec}-X^{1}∥}_{F}^{2}+{∥X^{rec}-X^{2}∥}_{F}^{2}\right) \end{array}$$

where *F* denotes the Frobenius norm. The structure loss calculates the structural similarity between input images and the reconstructed images

(14) $$\begin{array}{c} L_{str}=\frac{1}{2}\times (1-\mathrm{SSIM}(X^{rec},X^{1})+1-\mathrm{SSIM}(x^{rec},X^{2})) \end{array}$$

where SSIM denotes the structural similarity measure. With the value becoming larger, the input images and the reconstructed images have more similarity in structure.

For the labeled data, the supervised training loss is designed to calculate the cross entropy between predictions and labels, which enforces the network to learn class information from the labeled data. The detailed loss function is as follows:

(15) $$\begin{array}{c} L_{sp}=-\frac{1}{M+1}\sum_{i=1}^{\left(M+1\right)}\sum_{j=1}^{K}1\left[y=k\right]log(p\left(y_{j}|H\left(\omega_{i}\right)\right)) \end{array}$$

where *H* represents the multi-modal classifier with multiple full-connection layers. For the unlabeled data, the training loss is based on the semi-supervised contrastive learning strategy, which is demonstrated in the above section. Finally, the overall loss is as follows:

(16) $$\begin{array}{c} L=L_{sp}+\alpha L_{contr}+\beta \left(L_{div}+L_{mem}\right) \end{array}$$

where α and β denote loss weights of unsupervised contrastive learning loss and class-wise memory loss, respectively.

## 4. Experiments

### 4.1. Datasets

The proposed approach is evaluated on the VAIS dataset [38], which is the first publicly multi-modal maritime ship dataset. For now, as we know, it is also the only existing public multi-modal ship image dataset. The dataset comprises 2865 images (1623 visible ship images and 1242 infrared ship images) and includes six ship categories: cargo ships, middle–other ships, passenger ships, sailing ships, small boats, and tugboats. These images are collected at various times in one day, including dusk and dawn. Some images in the dataset are high-resolution while some images are blurred and hard to recognize. According to the paper [38], there are 1088 pairs of matched visible–infrared unregistered images. Other images are unpaired and low-resolution, including 154 night-time infrared images.

To evaluate the generalization ability of our approach, we use the Sentinel-1 dataset to test the model. The Sentinel-1 dataset [39] is collected from Sentinel-1 satellite single-looked complex (SLC) SAR images of imaging mode Stripmap. The Sentinel-1 SAR mission was launched by the European Space Agency (ESA), operating in C-Band with horizontal and vertical polarizations, providing SAR data in four different modes.

### 4.2. Comparison Methods

The comparison methods include the supervised learning baseline, a supervised ship classification method P2Net [40], pseudo-label-based baseline, and several state-of-the-art semi-supervised learning methods, including FixMatch [41], CoMatch [42], and Semicls [43]. Fixmatch [41] is a self-training method based on pseudo label generation technology that selects unsupervised samples with high-confidence pseudo labels and uses them as training targets. CoMatch [42] is a graph-based semi-supervised learning method based on the self-supervised learning framework, that generates pseudo labels based on image embeddings and learns better representations by graph-based contrastive learning. Semicls [43] is a semi-supervised contrastive learning method, that designs the class-aware contrastive module to explore reliable in-distribution data clustering and noisy out-of-distribution data contrasting. All the compared methods are trained under single-modal and multi-modal settings. SNL-DNLL [44] is a mutual learning framework based on pseudo-negative labels. In the single-modal setting, all methods take visible and infrared images as input, respectively. In the multi-modal setting, all methods utilize visible and infrared fused images as the input. For producing fused images, an image fusion model, nest-connection-based image fusion framework (NestFuse) [45], is used.

To verify the effectiveness of Semi-MMSC on the Sentinel-1 dataset, three methods (CNN, TL-CNN, and DSN), used in the paper [46], are selected as comparison methods. DSN can be viewed as the multi-modal classification method and combines spatial features and frequency features to classify targets.

### 4.3. Evaluation Indicators

For the evaluation of the proposed approach, four indicators are used: accuracy, precision, recall, and f1-score.

### 4.4. Implement Details

The network consists of two components, the local-level fusion module and the global-level interactive module. The local-level fusion module is based on the encoder–decoder architecture, which includes three parts: the local feature extractor, the local feature decoder, and the fusion layer. The local feature extractor is a convolutional network with one convolutional layer and four convolutional blocks. Each block contains two convolutional layers and one max-pooling operator. The convolutional layers all adopt 3 ∗ 3 kernel size and are equipped with the Relu activation function. The local feature decoder includes one convolutional layer and six convolutional blocks, which are connected by the nest connection architecture. The convolutional blocks of the decoder are made up of three convolution layers. During training, with the modal-specific encoder and modal-specific decoder with pre-trained loss, deep salient features of the two modalities are extracted, respectively. The fusion layer is built based on spatial and channel attention mechanisms, which are used to fuse these salient features of different modalities.

For mining the global-level correlation further, the global-level interactive module is built. The module consists of three parts: the global feature extractor, feature interactive module, and multi-modal classifier. The global feature extractor is based on ResNet architecture, which is used to extract global-level features. Then, these features are fed into the feature interactive module, which is equipped with three fully connected layers. Three fully connected layers include 400, 200, and 600 units, respectively, followed by a Batchnorm regularization and a Relu activation function. The input of the third fully connected layer is the interactive calculation results of the output of the second fully connected layer. Furthermore, the multi-modal classifier is built to predict the class probability of data points, which is equipped with two fully connected layers.

We adopt the Adam optimizer for optimization, and the learning rate is set to 0.0001. We train Semi-MMSC for 100 epochs to demonstrate its efficiency on the classification task and the batch size is set to 8. The time taken to complete one epoch of training is 110 s, and the duration for a single ship image inference is 0.6 s.

### 4.5. Dataset Processing

Because the sample number of each class on the VAIS dataset is imbalanced in practice, randomly sampled images with labels like standard semi-supervised learning methods are not class-balanced. For example, the tug boat class only has 50 samples. Hence, for conducting a thorough analysis of the proposed approach, the experiment under two kinds of label fraction settings is conducted in a class-balanced way, 10 samples of each class are used in the training, and a class-imbalanced way, 10% samples of each class are used. The experiment contains two parts, single-modal classification and multi-modal classification. Specifically, in single-modal learning, the experiment takes images of two modalities separately as inputs, which are represented by ‘vis’ and ‘inf’, respectively. The classification results of all compared methods on the VAIS dataset are shown in Table 2.

The experimental visible and infrared images are preprocessed with the unified data augmentation technology, including image scaling, image color jitter, and the direction adjustment of the ship image via horizontal transform. The experimental SAR images are preprocessed using the method in [40]. All experiments are performed on Intel(R) Xeon(R) Gold 5115 CPU @ 2.40GHz (Intel, Santa Clara, CA, USA) and NVIDIA Tesla V100 (NVIDIA, Santa Clara, CA, USA) and are implemented in Python 3.7 using the PyTorch framework.

### 4.6. Experiment Results

It is observed that deep semi-supervised learning methods based on pseudo labels do not show better performance than the supervised learning baseline, which proves that simply generating pseudo labels for the unlabeled data is not effective. Specifically, many methods achieve the same identical accuracy value as supervised learning, like 32.44% on the first label fraction setting. The reason for this problem should be that a large number of unlabeled samples can confuse the model and cause model overfitting so the model can only classify a few supervised samples. Semi-supervised learning methods show better performance than the supervised baseline and pseudo-label baseline on the VAIS dataset. It indicates that making use of unlabeled data and limited data appropriately is effective in improving the semi-supervised classification performance.

By comparing results under the class-balanced (10 samples of each class) and class-unbalanced (10% samples of each class) conditions, it is noted that a balanced sample size for each category is more conducive to the model learning class information, since most methods show better performance under the class-balanced condition.

Furthermore, some multi-model classification methods show worse performance than single-model classification methods, which indicates that simply concatenating features of different modalities can not learn complementary ship hull and superstructure information effectively, and redundant category independent information from different modalities causes the model performance degradation.

Comparing existing methods, the proposed approach (Semi-MMSC) obtains a top-1 accuracy of 63.69% with 10 samples per class, which achieves a 15.6% improvement. On the condition of 10% samples per class, the performance of Semi-MMSC can further improve to 72.05%. In addition, Semi-MMSC obtains the top-5 accuracy of 100% on two semi-supervised settings. Overall, Semi-MMSC produces ship class predictions that are more confident and accurate in the semi-supervised multi-modal setting.

As shown in Table 3, for evaluating the generalization ability, the proposed approach (Semi-MMSC) conducts classification experiments on the Sentinel-1 dataset. Semi-MMSC adopts the same architecture of DSN [46] to extract frequency features as texture features and then builds the global-level interactive module based on these features to learn cross-modal correlation. Furthermore, the semi-supervised contrastive learning strategy is imported. Semi-MMSC achieves better performance than other methods, which obtains an average accuracy of 93.90% and an average f1-score of 0.938.

**Figure 3:**
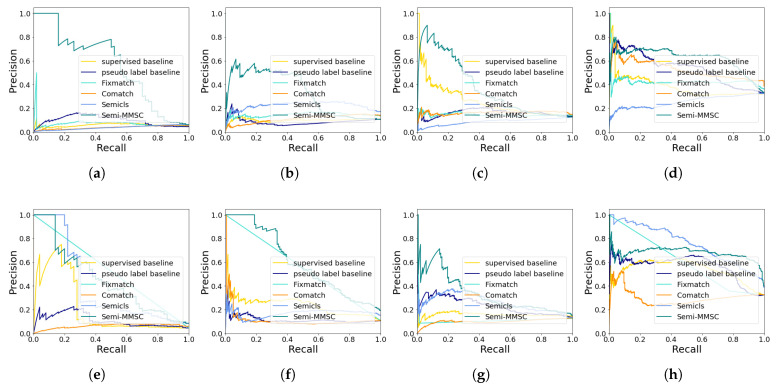
precision–recall curves of our approach and other competitors on two label fraction settings: (**a**–**d**) are the precision–recall curves of the classes ‘tug boat’, ‘medium passenger ship’, ‘medium other ship’, and ‘small boat’ on the first label fraction setting, respectively; (**e**–**h**) are the precision–recall curves of the classes ‘tug boat’, ‘medium passenger ship’, ‘medium other ship’, and ‘small boat’ on the second label fraction setting.

The precision–recall curves of our proposed approach and other competitors respectively on two label fractions settings are demonstrated in Figure 3. We compare Semi-MMSC with some state-of-the-art semi-supervised learning methods (i.e., Comatch and Semicls) and some fusion methods. From the results, it is observed that the proposed Semi-MMSC significantly outperforms other methods in four different individual classes (the class ‘small boats’ with the most samples, the class ‘tug boats’ with the fewest samples, the class ‘medium passenger ship’ and the class ‘medium other ship’) at the first label fraction setting. It is noted that the class ‘medium passenger ship’ and the class ‘medium other ship’ are difficult to distinguish. On the second label fraction setting, Semi-MMSC is inferior to Semicls and Fixmatch on the class ‘small boat’, which can be attributed to the fact that these methods may only focus on the difference of the ship shape, like small and medium. On the class ‘tug boat’, the break-even point of Semi-MMSC and Semicls is roughly equal. In general, Semicls and Fixmatch have poor performance on other classes, especially when few samples are obtained. Overall, Semi-MMSC demonstrates the robustness of the semi-supervised multi-modal ship classification task.

**Figure 4:**
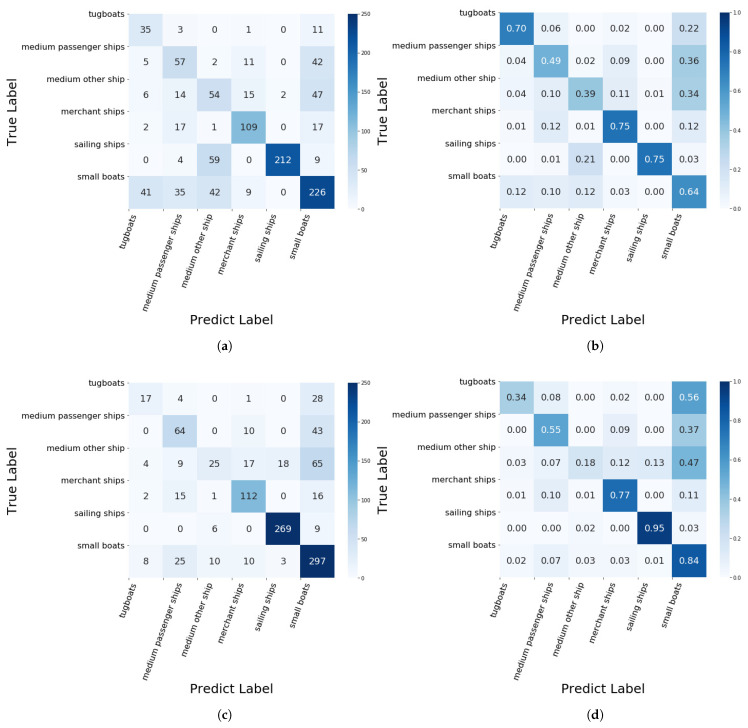
The confusion matrices of the proposed approach on two label fraction settings, (**a**,**c**) denote the confusion matrices of correct sample numbers of each class; (**b**,**d**) denote the confusion matrices of the class specific accuracy.

The confusion matrices of the proposed approach on the VAIS dataset are depicted in Figure 4. As displayed in Figure 4a, the diagonal elements of the confusion matrix denote the correct sample numbers of each class under the first label fraction setting. The class specific accuracy (%) is shown in Figure 4b, where the major confusion occurs between class medium passenger ships and class medium ‘other’ ships. We observe that images of these two classes are similar to each other and the sample number of two classes is also close. In addition, the confusion matrices under the second label fraction setting are shown in Figure 4c,d, the class specific accuracy of each class becomes also imbalanced, classes with a large amount of samples have higher accuracy (i.e., small boats, sailing ships) and classes with few samples have lower accuracy (i.e., tug boats).

### 4.7. Ablation Study

An extensive ablation study is performed to verify the effectiveness of different components in the proposed approach.

**Effectiveness of Multi-level Components:** The proposed approach performs the cross-modal feature correlation to learn complementary information. To prove the effectiveness of local-level and global-level components, the corresponding experiment is designed. The local-level fusion module only simply concentrates features of different modalities based on the pix-level reconstruct loss. The global-level interactive module implements the feature interaction among the global features of the two modalities with local-level fused features as a medium. As shown in Table 4, comparing the employment of only local-level feature fusion, the proposed approach achieves 57.26% and 65.99% on the Top-1 accuracy metric, respectively, on two label fraction settings. Based on the multi-level cross-modal interactive architecture, Semi-MMSC further improves Top-1 accuracy by 6.43% and 4.87%.

**Effectiveness of Different Backbones:** To prove the robustness of the ResNet backbone, we select the transformer architecture [47] as the backbone and conduct the comparison experiment, which is shown in Table 5. Compared with the ResNet backbone, the transformer backbone increases Top-1 accuracy under the first label fraction setting by 13.6949% while decreasing Top-1 accuracy under the second label fraction setting by 2.9412%. It is worth noting that the proposed approach can outperform the previous methods no matter which backbone is chosen. Meanwhile, the ResNet backbone can capture more detailed information directly from input images while the transformer backbone relies on labeled samples to learn global semantic information. This indicates that the transformer architecture captures multidimensional information such as visual features, spatial structure, and styles of images, while ResNet captures important information such as shape, texture, objects, and scene in images through convolutional neural networks. When more labeled samples are provided for training, the transformer can exhibit better performance. For adapting the condition of a few samples, we select the ResNet as the backbone.

Then, we give an in-depth analysis of our design for the semi-supervised contrastive learning strategy, and demonstrate the benefits of them through comparative experiments.

**Effectiveness of Contrastive Representations:** Table 6 provides some results about self-supervised contrastive learning strategy. If we train the model with only pseudo label learning to predict unlabeled training data, we can obtain Top-1 accuracy of 39.61% and 44.39%. If we use the contrastive head module in the feature space, Top-1 accuracy improves by 18.65% and 25.09%. In other words, pseudo-label-based self-supervised learning demonstrates poor classification performance, which is the motivation of the self-supervised contrastive learning strategy. We believe this will help the model learn class-wise complementary information during training, and it can be seen that our method gains 5.42% and 2.57% benefits with the whole function from the results reported in Table 5.

**Memory Feature Selection Strategy:** In this part, we discuss the impact of different memory selection strategies. Three alternative sample selection methods are listed as follows:

- using correlated features of different modalities as the input of the memory mechanism, including $\omega^{1}$, $\omega^{2}$, and $\omega^{f}$.
- using the pair-wise features, including three pairs ($\omega^{1}$, $\omega^{f}$), ($\omega^{2}$, $\omega^{f}$), and ($\omega^{1}$, $\omega^{2}$).
- using the feature similarity representations, which are the output of the contrastive head.

As shown in Table 7, selecting the combination of features (i.e., ($\omega^{1}$, $\omega^{f}$)) performs even worse than the single-modal feature. The reason is that local-level fused features have much texture information and less semantic information, which shows that the simple combination between features of different modalities has negative effects on performance. Additionally, using contrastive representations is superior to using similarity representations by 7.16% and 5.51%, which demonstrates that the former can store more class information.

**Figure 5:**
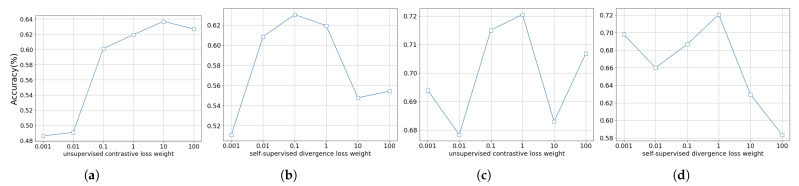
The analysis of default hyper-parameters on two different label fraction settings, unsupervised contrastive loss weight α and self-supervised divergence loss weight β. (**a**,**c**) Varying weight α, which controls the unsupervised contrastive learning. (**b**,**d**) Varying weight β for the self-supervised learning.

**Evaluation of Weight Assignment for Loss Functions:** We analyze the impact of several hyper-parameters, which are, respectively, unsupervised contrastive loss weight α and self-supervised divergence loss weight β. The values of loss weights are selected from 0.001, 0.01, 0.1, 1, 10, 100 correspondingly, and the results are shown in Figure 5. The two losses are both the main optimization objective as they determines the quality of unsupervised learning and have an important impact on the final prediction. It can be observed that the optimal performance can be obtained when the values of (α, β) are set to (10, 1) and (1, 1), respectively, on two different label fraction settings.

## 5. Conclusions

In this paper, we propose a novel Semi-supervised Multi-Model Ship Classification approach, which consists of the multi-level interactive network and semi-supervised contrastive learning strategy. The multi-level cross-modal interactive network encoders deep correlated features containing cross-modal complementary information in a multi-level feature interaction manner. Then, for taking advantage of unlabeled data in the training stage, the semi-supervised contrastive learning strategy is designed to enrich supervised signals of unlabeled data to guide the network training. Extensive experiments on the public datasets demonstrate that our approach obtains a state-of-the-art performance. In the future, we will focus on applying this approach in the field of the fishery industry and further extend the research by designing more refined feature extraction modules like transformers.

## Figures and Tables

**Table 1:** The architecture of the local-level fusion module.

Name	Type	Output Size
ConvEn_1	Conv + Relu	(16, 224, 224)
ConvEn_2	Conv + Relu	(8, 224, 224)
	Conv + Relu	(64, 224, 224)
	MaxPooling	(64, 112, 112)
convEn_3	Conv + Relu	(32, 112, 112)
	Conv + Relu	(112, 112, 112)
	MaxPooling	(112, 56, 56)
convEn_4	Conv + Relu	(56, 56, 56)
	Conv + Relu	(160, 56, 56)
	MaxPooling	(80, 28, 28)
convEn_5	Conv + Relu	(40, 28, 28)
	Conv + Relu	(208, 28, 28)

**Table 2:** Accuracy for the VAIS dataset on two different label fraction settings.

	Methods	Top-1 Accuracy Label Fraction (%)		Top-5 Accuracy Label Fraction (%)	
		**10 Samples** **per Class**	**10% Samples** **per Class**	**10 Samples** **per Class**	**10% Samples** **per Class**
single-modal classification	supervised baseline (vis)	32.4449	26.1029	95.4044	89.2463
	supervised baseline (inf)	40.5331	56.6176	88.0515	97.0588
	pseudo label baseline (vis)	32.4449	32.4449	73.8971	95.4044
	pseudo label baseline (inf)	32.4449	32.4449	86.5809	86.5809
	Fixmatch [41] (vis)	29.3199	26.1029	67.5551	95.4044
	Fixmatch [41] (inf)	32.7206	34.5588	95.4044	70.6801
	Comatch [42] (vis)	46.2316	49.5404	98.6213	94.2096
	Comatch [42] (inf)	48.0699	35.2941	95.4044	89.2463
	Semicls [43] (vis)	32.4449	57.0772	73.8971	99.6324
	Semicls [43] (inf)	32.4449	32.4449	89.2463	87.3162
	SNL-DNLL [44] (vis)	12.6838	26.1029	67.5551	86.5809
single-modal classification	SNL-DNLL [44] (inf)	10.7537	32.3420	73.8971	86.5809
	P2Net [40] (vis)	32.4449	26.1029	95.4044	89.2463
	P2Net [40] (inf)	40.5331	56.6176	88.0515	97.0588
multi-modal classification	supervised fusion baseline	34.3750	54.5037	89.9816	93.6581
	pseudo label fusion baseline	39.6140	44.3934	77.7574	94.7610
	Fixmatch [41]	34.8346	32.4449	95.4044	87.3162
	Comatch [42]	32.4449	33.1801	95.4044	95.4044
	Semicls [43]	32.4449	32.4449	95.4044	73.8971
	Semi-MMSC	**63.6949**	**72.0588**	**100.00**	**100.00**

**Table 3:** Generalization experiments on the Sentinel-1 dataset.

	Accuracy				F1-Score			
**Classes**	**Semi-MMSC**	**DSN [46]**	**TL-CNN [46]**	**CNN [46]**	**Semi-MMSC**	**DSN [46]**	**TL-CNN [46]**	**CNN [46]**
Forest	93.75 ± 1.76	98 ± 2.74	**99.50 ± 1.12**	96.50 ± 1.37	**0.9677 ± 0.009**	0.958 ± 0.011	0.959 ± 0.011	0.949 ± 0.033
Water	**100 ± 0**	100 ± 0	100 ± 0	100 ± 0	**1 ± 0**	1 ± 0	1 ± 0	1 ± 0
Agriculture	**100 ± 0**	93.50 ± 2.24	94.50 ± 2.09	95.00 ± 3.06	**0.969 ± 0.008**	0.959 ± 0.014	0.96 ± 0.018	0.955 ± 0.023
Industrialbuilding	**95 ± 0**	90 ± 5.59	81 ± 8.77	79.50 ± 4.47	**0.95 ± 0**	0.907 ± 0.046	0.832 ± 0.065	0.804 ± 0.052
Residential	**100 ± 0**	100 ± 0	97.50 ± 3.06	94.00 ± 5.18	0.987 ± 0	**0.990 ± 0.010**	0.958 ± 0.014	0.926 ± 0.037
Skyscraper	**100 ± 0**	92.00 ± 3.26	91.50 ± 7.42	89.00 ± 5.76	**1 ± 0**	0.887 ± 0.029	0.844 ± 0.025	0.813 ± 0.045
Storagetank	85 ± 0	**85.50 ± 6.71**	82.00 ± 4.11	72.50 ± 6.85	**0.877 ± 0.007**	0.858 ± 0.035	0.817 ± 0.036	0.741 ± 0.053
Container	**85.25 ± 3.88**	84.50 ± 4.11	73.50 ± 10.98	69.50 ± 9.25	**0.88 ± 0**	0.873 ± 0.023	0.811 ± 0.075	0.762 ± 0.0457
Average	**93.90 ± 0.21**	92.94 ± 1.05	89.94 ± 1.75	87.00 ± 2.01	**0.938 ± 0.002**	0.929 ± 0.011	0.898 ± 0.019	0.869 ± 0.022

**Table 4:** Effectiveness of local-level and global-level components.

Methods	10 Samples per Class		10% Samples per Class	
	**Top-1 acc**	**Top-5 acc**	**Top-1 acc**	**Top-5 acc**
Local-level fusion	57.2610	100.00	65.9926	100.00
Global-level fusion	55.2390	100.00	67.1875	100.00
Semi-MMSC	63.6949	100.00	72.0588	100.00

**Table 5:** Effectiveness of different backbones.

Backbones	10 Samples per Class		10% Samples per Class	
	**Top-1 acc**	**Top-5 acc**	**Top-1 acc**	**Top-5 acc**
Transformer	50.0000	100.00	75.0000	100.00
ResNet	63.6949	100.00	72.0588	100.00

**Table 6:** The influence of different semi-supervised contrastive learning modules based on the VAIS dataset.

Adding Module	Top-1 Accuracy on the First Label Fraction	Top-1 Accuracy on the Second Label Fraction
baseline	39.6140	44.3934
add contrastive head module	58.2721	69.4853
add memory module	63.6949	72.0588

**Table 7:** Impact of different memory feature selection strategy on the model.

Feature Selection Strategy	Accuracy (%)	
	**10 Samples** **per Class**	**10% Samples** **per Class**
using single-modal features	56.0662	63.2353
	52.2978	64.3382
	59.2831	65.0397
using pair-wise features	54.4118	61.3971
	52.2978	64.1544
	51.6544	65.6541
using the feature similarity representations	56.5257	62.0404
	53.9522	62.9596
	55.5147	66.5441
using contrastive representations	63.6949	72.0588

## Data Availability

The data presented in this study are available on request from the corresponding author.
